# Erythropoietin treatment in murine multiple myeloma: immune gain and bone loss

**DOI:** 10.1038/srep30998

**Published:** 2016-08-02

**Authors:** Naamit Deshet-Unger, Sahar Hiram-Bab, Yasmin Haim-Ohana, Moshe Mittelman, Yankel Gabet, Drorit Neumann

**Affiliations:** 1 Department of Cell and Developmental Biology, Sackler Faculty of Medicine, Tel Aviv University, Israel; 2 Department of Anatomy and Anthropology, Sackler Faculty of Medicine, Tel-Aviv University, Israel; 3 Department of Medicine, Tel Aviv Sourasky Medical Center, Sackler Faculty of Medicine, Tel-Aviv University, Israel

## Abstract

Multiple myeloma (MM) is a plasma cell malignancy, characterized by osteolytic lesions and monoclonal immunoglobulins. The anemia, accompanying the disease is often treated with recombinant human EPO. Diverse non-erythropoietic effects of EPO have led us to question its combined action on the immune system and bone in the 5T33MM mouse model. EPO administration to MM mice attenuated disease progression as demonstrated by a decrease in serum MM IgG2b, splenic CD138 expressing cells, IL-6 and RORγτ transcripts in bone marrow (BM). IFN-γ transcript levels and macrophages (F4/80^+^CD11b^+^) in the BM both increased ~1.5 fold in the EPO-treated MM mice. *In-vitro*, EPO stimulated phagocytosis of 5T33MM cells (+30%) by BM-derived macrophages. In contrast, high-resolution microCT analysis of distal femurs revealed EPO-associated bone loss in both healthy and 5T33MM mice. EPO significantly increased expression of the osteoclastogenic nuclear factor-kappa B ligand (RANKL) in healthy mice, but not in MM mice, likely due to antagonizing effects on MM progression. Thus, in MM, EPO may act as a double-edged-sword stimulating immune response, while accelerating bone resorption, possibly *via* direct action on BM macrophages. This study supports a prudent approach of treating anemia in MM patients, aiming to maintain EPO-associated anti-MM effects, while considering bone damage.

Multiple myeloma (MM) is a plasma cell malignancy and the second most common hematologic cancer. It presents a broad range of clinical symptoms, including clonal expansion of plasma cells in the bone marrow (BM) and monoclonal immunoglobulins in blood and urine^1^. Two thirds of MM patients suffer from anemia and part of these patients are treated with recombinant human erythropoietin (rHuEPO) e.g.^2^. Erythropoietin (EPO), produced in the kidney, is a crucial hormone that regulates the production of red blood cells^3^. It exerts its effects by binding to its receptor (EPO-R) expressed on erythroid progenitors in the BM, leading to their expansion, differentiation and/or survival^4^. Beyond its erythropoietic activity, EPO was suggested to act as a tissue protective factor, notably in cardiac and neuronal tissues^5^. Several studies, including our own, have reported that the immune system is also a target for EPO^6^,^7^,^8^,^9^,^10^,^11^,^12^,^13^,^14^,^15^,^16^. We have previously reported that macrophages and dendritic cells express functional EPO-Rs^9^,^10^,^16^, thus pointing to these cells as likely candidates for mediating EPO effects on the immune system. We^6^,^7^,^11^,^17^ and others^18^,^19^, have noted prolonged survival and improved immunological functions in MM mouse models and MM patients, linked to EPO administration. Notably, others reported contradicting data regarding rHuEPO treatment in MM patients^20^,^21^, which warrants further research to elucidate this question.

In MM, the proinflammatory cytokine interleukin-6 (IL-6) plays a critical role. It is secreted by the MM plasma cells as well as by the BM stromal cells and plays a critical role in MM progression^22^. Controversies exist regarding Th17 and Treg cell levels and function in MM patients^23^. Most often, MM patients display elevated Treg and Th17 cells^23^. The levels of Treg cells were shown to increase in MM patients along with MM progression and often indicate a higher risk disease^24^.

Myeloma bone involvement is a common manifestation of the disease, affecting more than 80% of patients^25^. Bone pain, pathological fractures, lytic lesions and other bone problems are common. Imbalanced bone remodeling in the myeloma BM is caused by increased osteoclast activity, together with reduced osteoblast function. MM cells homing to the BM are believed to exert a major catabolic effect mediated by various interactions with stromal cells, leading to recruitment, differentiation and activation of osteoclast progenitors within the BM and inhibition of osteogenesis^25^,^26^. The crosstalk between the hematological/immune and bone systems in MM and their response to EPO treatment are, as yet, not completely resolved.

Monocyte differentiation into osteoclasts (the bone resorbing cells) is driven and regulated by the receptor activator for nuclear factor kappa B (RANK)/RANK ligand (RANKL)/osteoprotegerin (OPG) axis^27^. RANKL is the main pro-osteoclastogenic cytokine, and it is antagonized by OPG. Myeloma plasma cells express RANKL and induce an imbalance in the RANKL/OPG interactions, resulting in increased osteoclastic activation and bone resorption^25^,^26^. EPO effects on bone may depend on pathophysiological conditions. EPO supported bone formation in fracture healing models e.g.^28^, while, it induced bone loss in adult mice^29^,^30^,^31^. Our recent findings that EPO directly stimulates bone loss *via* activation of EPO-R signaling in the monocytic lineage^30^, coupled with the central role of macrophages in MM^32^, highlight the need to determine EPO effect on bone in the context of MM.

The 5T33MM mouse model originates from spontaneously developed MM in elderly mice of the C57BL/KalwRij strain^33^. The clinical characteristics of this model, including the selective localization of the MM cells in the BM and elevated serum monoclonal immunoglobulin IgG2b Kappa (IgG2bκ), are similar to those of human myeloma^34^,^35^, rendering it a useful model for studying MM and relevant therapeutic approaches. The absence of severe bone disease in the 5T33 MM model^36^,^37^ serves well the purpose of testing EPO effects on bone. It allows separation of the bone disease due to MM from the EPO effects. One can easily conclude what would happen in patients with MM displaying bone diseases who are treated with EPO for their anemia.

Here we show that in 5T33MM, EPO acts as a double-edged sword, by improving immune parameters on one hand, yet accelerating bone resorption on the other.

## Results

### EPO treatment in 5T33MM mice alleviates anemia and attenuates IgG2b, plasma cells and IL-6

The 5T33MM mouse disease model is created by injection of 5T33MM cells into wildtype C57BL/KalwRij mice^33^. To assess the effects of EPO treatment in the 5T33MM mouse model on anemia, we initially measured hemoglobin (Hgb) levels, which as expected were reduced in the MM mice. EPO treatment corrected Hgb levels in the MM mice and elevated Hgb levels in the healthy mice (Figure S1).

**Figure 1:**
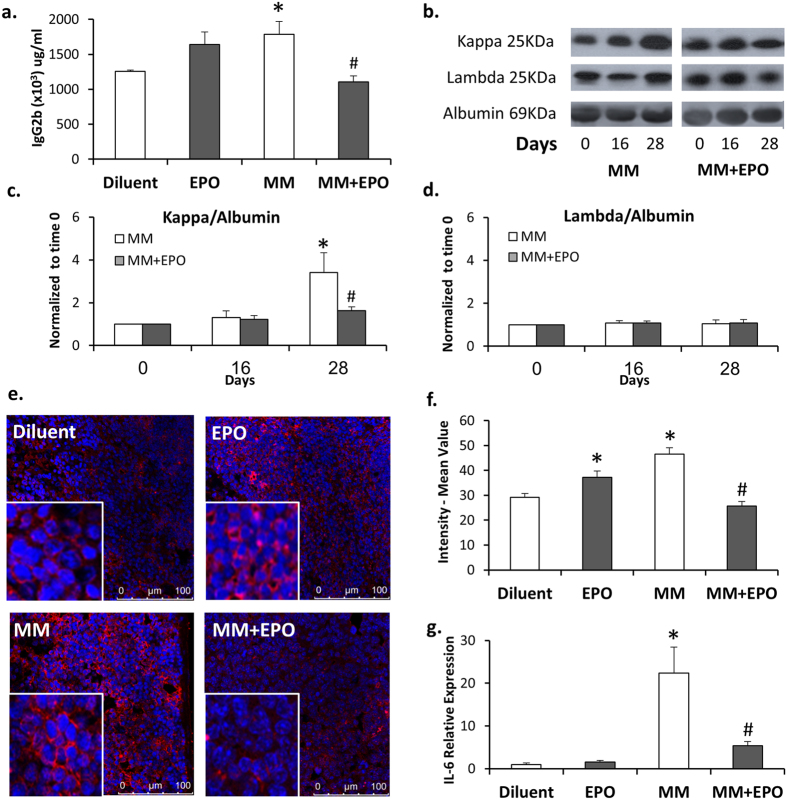
EPO treatment attenuates disease progression. Sera from healthy or MM mice treated with EPO or diluent were subjected to **(a)** ELISA analysis for IgG2b on day 28 **(b)** Representative Western blot probed with anti-kappa, anti-lambda or anti-albumin antibodies. **(c–d)** Graphs represent a summary of all mice analyzed N>7. Data, Mean ± SEM, were analyzed by Student’s *t*-test *MM day 28 *versus* MM day 0; ^#^MM + EPO *versus* MM on day 28, p < 0.05. **(e)** Splenic sections were stained using a fluorescent antibody directed to the CD138 (red staining) surface marker, nuclei were stained with DAPI (blue). Representative images are shown. **(f)** Graphs represent a quantitation of at least 3 fields of sections from N = 3 mice in each group. **(g)** RT-PCR quantification of IL-6 mRNA expression in BM from healthy or MM mice treated with EPO or diluent. Transcript levels in diluent-injected mice were considered as 1, N > 7. Data in a, f and g, Mean ± SEM, were analyzed by 1-way ANOVA with Bonferroni post-hoc test *MM *versus* Diluent; ^#^MM + EPO *versus* MM, p < 0.05.

We then analyzed serum levels of IgG2b, which correlate with disease progression^34^,^35^. As shown in Fig. 1a, diluent-injected MM mice displayed a 42% (p = 0.038) increase in the levels of IgG2b compared to those measured in their healthy counterpart mice. Treatment of 5T33MM mice with EPO was associated with normalized levels of serum IgG2b (38% decrease, p = 0.005), approaching those of healthy, diluent-injected control mice. Note that in healthy mice, EPO injection led to a 30% increase, in the levels of serum IgG2b. Although not statistically significant, this trend is in line with our previous findings on EPO-mediated improved immunological functions^7^,^8^,^9^,^10^,^13^. The 5T33MM mouse model is characterized by secretion of monoclonal IgG2bκ. Hence, we validated by Western blot analysis the increase in the pathological IgG2bκ and its subsequent reduction by EPO in the 5T33MM mice. We thus determined by Western blot analysis the levels of kappa and lambda light chain in the sera of the mice (Fig. 1b–d). The levels of the kappa light chains at day 16 following 5T33 cells’ inoculation were comparable to those measured at day 0, and were increased after 28 days. Notably, the levels of the lambda light chain remained constant at all-time points. EPO treatment in the 5T33MM mice resulted in a 50% (p < 0.05) decrease in the kappa light chain (Fig. 1b–d), in accordance with the ELISA quantification of the IgG2b (Fig. 1a). Moreover, EPO injection to the 5T33MM mice led to a 46% (p < 0.001) reduction of splenic CD138 expressing cells (plasma cells) compared to the diluted-injected 5T33MM mice. EPO-treated healthy mice displayed a 27% (p = 0.05) increase in the levels of splenic CD138 expressing cells as compared to the diluent-injected healthy mice (Fig. 1e–f). Of note, we measured the levels of IgA in the sera of the mice, as a reference of non-MM immunoglobulin. The levels of IgA were also reduced in MM mice but were not affected by EPO (Figure S2), suggesting that EPO treatment reduced the levels of the pathological immunoglobulin, but not of the normal ones.

IL-6 levels are increased in MM and may thus serve as a surrogate marker for MM disease progression^22^. In that respect, and in line with our above findings on EPO-associated decrease in the IgG2b (Fig. 1a), we found a 4-fold decrease in transcript levels of IL-6 in the BM of EPO-treated MM animals (Fig. 1g).

These findings show that EPO treatment attenuated 5T33MM disease progression as determined by a reduction in serum IgG2b, splenic plasma cells and IL-6 transcripts in the BM.

### EPO treatment decreases RORγτ, but not FOXP3 in 5T33MM mice

**Figure 2:**
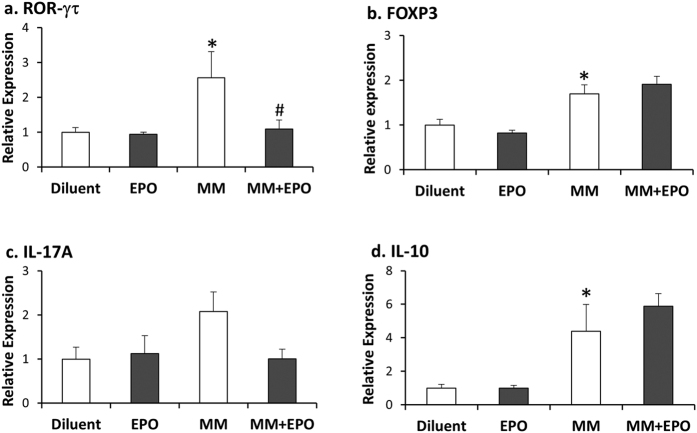
EPO treatment decreases RORγτ, but not FOXP3 in MM mice. RT-PCR quantification of **(a)** RORγτ **(b)** FOXP3 **(c)** IL-17A and **(d)** IL-10 mRNA expression in BM from healthy and MM diluent or EPO-injected mice. Transcript levels in diluent-injected healthy mice were considered as 1, N > 6. Data, Mean ± SEM, were analyzed by 1-way ANOVA with Bonferroni post-hoc test, *versus Diluent; ^#^*versus* MM, p < 0.05.

Th17 and Treg cells are considered prognostic markers in MM, as they are often elevated in MM patients^23^. Here we addressed Treg and Th17 associated parameters in the 5T33MM mouse model. In the BM of diluent-administered MM mice we found increased levels of both RORγτ and FOXP3, the hallmark transcription factors of Th17 and Treg cells, respectively, as compared to healthy mice (2.9-fold and 1.6-fold, respectively) (Fig. 2a,b). The transcript levels of IL-17A, the Th17-associated cytokine (Fig. 2c), displayed the same trend as RORγτ although not statistically significant. In line with these results, we also found an elevation in transcript levels of the Treg-associated cytokine IL-10 in BM of MM compared to diluent mice (~5-fold) (Fig. 2d). EPO treatment in the MM mice was associated with reduced expression of RORγτ and IL-17A (−65% and −50%, p = 0.006 and p = 0.3, respectively), as compared to their counterpart, diluent-treated MM mice (Fig. 2a,c). Notably, EPO treatment in MM mice did not reduce the transcript levels of FOXP3 and IL-10 (Fig. 2b,d).

Our findings demonstrate an EPO-mediated decrease in the levels of RORγτ in MM mice that are indicative of MM progression.

### EPO-associated increase in BM macrophages and interferon (IFN)-γ mRNA expression in MM mice

**Figure 3:**
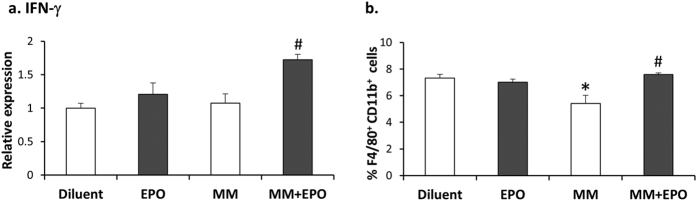
EPO associated increase in IFN-γ mRNA expression and macrophage population in BM of MM mice. **(a)** RT-PCR quantitation of IFN-γ mRNA expression in total BM from healthy and MM diluent or EPO-injected mice. Transcript levels in diluent-injected mice were considered as 1, N = 8–10 mice in each group. **(b)** Flow cytometry analysis representing %F4/80^+^CD11b^+^ cells from total BM of diluent or EPO-injected, healthy and MM mice. N = 7–12 mice in each group. Data, Mean ± SEM, were analyzed by 1-way ANOVA with Bonferroni post-hoc test, *versus Diluent; ^#^*versus* MM, p < 0.05.

IFN-γ is one of the major Th1-associated cytokines in MM^38^. EPO action on IFN-γ levels in the BM of 5T33MM mice was determined by RT-PCR. EPO administration in 5T33MM mice was associated with a 60% increase (p = 0.003) in IFN-γ transcript levels as compared to diluent-treated 5T33MM mice (Fig. 3a). EPO treatment in the 5T33MM mice also resulted in a 38% (p = 0.012) increase in the level of BM macrophages expressing F4/80^+^CD11b^+^ as compared to diluent treated MM mice (Fig. 3b). Notably, EPO did not affect the levels of F4/80^+^CD11b^+^ cells in the BM of healthy mice, in contrast to the EPO-mediated elevation of these cells observed in the spleen^10^.

### EPO up-regulates phagocytosis of 5T33MM cells by bone marrow derived macrophages (BMDM) *in vitro*

**Figure 4:**
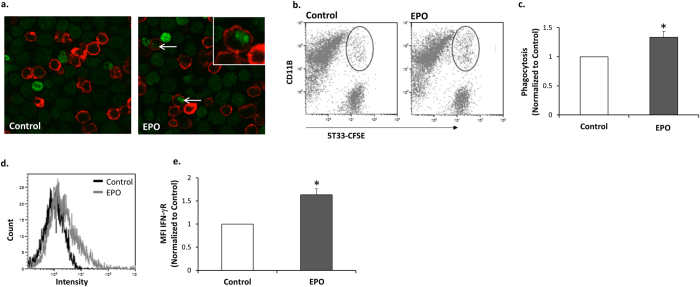
EPO up-regulates phagocytosis of 5T33MM cells by BMDM, and increases surface IFN-γR on BMDM. **(a)** BMDM were cultured ±EPO (5 U/ml) for 24 h. CFSE-labeled 5T33MM cells (green) were added to CD11b-labeled BMDM (red) for 24 h. Confocal images x40 magnification. Arrows = phagocytosed MM cells. **(b)** The same cultures were subjected to Flow cytometry analysis for CD11b (PE label) and CFSE-labeled 5T33MM cells. Phagocytosis was determined as the number of cells positive for both CFSE and CD11b. **(c)** Summary of 5 independent experiments; as described in b, data are Mean ± SEM; *versus Diluent, p < 0.05. **(d)** BMDM were cultured ± EPO (5 U/ml) for 24 h. Cells were labeled with anti CD11b and anti IFN–γR. The plot depicts a representative histogram, black and grey lines represent control and EPO treatments, respectively. **(e)** Summary of 3 independent experiments. MFI of the flow cytometry analysis was calculated. Data, Mean ± SEM, were analyzed by Student’s *t*-test, *versus Control, p < 0.05.

We have previously demonstrated that BMDM that are generated *in vitro* from whole BM, harbor functional EPO-Rs and that EPO-treated BMDM displayed enhanced phagocytic activity of E. coli bacteria^10^. We thus examined whether EPO has a direct effect on the capacity of BMDM to phagocytose MM cells. Cultured BMDM subjected to EPO treatment *in vitro* for 16 hours displayed a 33% (p = 0.01) increase in phagocytosis of 5T33MM cells (Fig. 4a–c). The enhanced capacity of the EPO treated BMDM to phagocytose the 5T33MM cells was accompanied by a 63% (p < 0.0001) increase in IFN-γR levels on the cell surface of the BMDM (Fig. 4d,e).

Hence, EPO treatment in 5T33MM mice leads to increased IFN-γ levels and macrophages (F4/80^+^CD11b^+^ cells) in the BM and enhanced phagocytosis of the 5T33MM cells *in vitro* by BMDM.

### EPO-driven bone loss in healthy and in 5T33MM mice

**Figure 5:**
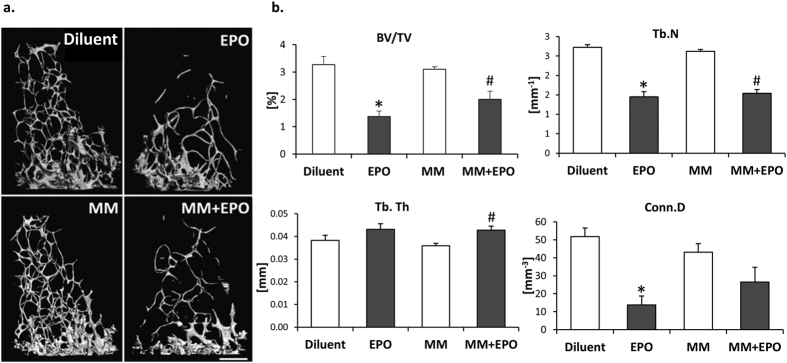
EPO injections induce bone loss in healthy and in 5T33MM mice. **(a)** μCT images of distal femoral trabecular bone of diluent or EPO-injected, healthy and MM mice. **(b)** BV/TV - trabecular bone volume density, Conn.D - connectivity density, Tb.N - trabecular number, Tb.Th - trabecular thickness, N > 4 mice per group. Data, Mean ± SEM, were analyzed by 1-way ANOVA with Bonferroni post-hoc test, *versus Diluent; ^#^*versus* MM, p < 0.05.

Osteolysis is a common complication of MM^25^. Considering that macrophages are the cellular precursors of osteoclasts and our recent demonstration that EPO induces bone loss by increasing osteoclastogenesis both *in vivo* and *in vitro*^30^, we questioned the effect of EPO on bone metabolism in the context of MM. Similar to our previous observation in healthy mice, in the 5T33MM mouse model we found that EPO-treated MM mice display a ~40% decrease in trabecular bone volume fraction (BV/TV) in the distal femoral metaphysis compared to diluent-injected MM mice (Fig. 5a,b). This was associated with a reduction in trabecular number (Tb.N) and connectivity density (Conn.D), but not in the mean trabecular thickness (Tb.Th). Of note, the bone μCT scans of MM mice did not display bone loss, in comparison to diluent-injected mice. This is in line with previous reports^36^,^37^, demonstrating a paucity of evident osteolytic lesions as well as the absence of generalized osteoporosis in the 5T33MM mouse model.

### EPO treatment affects RANKL/OPG mRNA ratio in BM of 5T33MM mice

**Figure 6:**
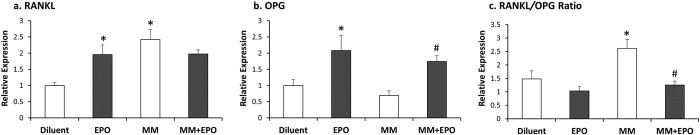
EPO treatment affects RANKL/OPG mRNA ratio in BM of 5T33MM mice. **(a)** RANKL and **(b)** OPG **(c)** RANKL/OPG ratio expression in BM of diluent- or EPO-injected healthy and MM mice, normalized to house-keeping Hprt mRNA and relative to diluent-treated healthy mice, considered as 1. Mean ± SEM of 6–8 mice were analyzed by 1-way ANOVA with Bonferroni post-hoc test, *versus Diluent; ^#^*versus* MM, p < 0.05.

To gain insight into the effect of EPO on bone in the MM mice, we analyzed the expression level of RANKL and OPG in the BM of EPO-treated, healthy and MM mice. In the 5T33MM mice, the disease resulted in a significant increase in RANKL levels, as compared to their healthy counterparts (p = 0.001, Fig. 6a), although it did not translate into detectable bone loss *in-vivo* (Fig. 5). In line with the observed bone loss (Fig. 5), healthy mice treated with EPO exhibited increased expression levels of RANKL transcripts. However, in MM mice, EPO treatment did not further increase the already elevated RANKL levels in comparison to their diluent injected controls (Fig. 6a). Surprisingly, the levels of OPG (Fig. 6b) and that of the calculated RANKL/OPG ratio^39^ (Fig. 6c), were exactly opposite to what would be expected from the bone loss observed in our μCT analysis (Fig. 5). Namely, OPG levels were higher in the EPO-treated osteoporotic mice than in the diluent-treated healthy and MM mice which do not show a bone loss.

Collectively, our data indicate that EPO may limit MM progression by modulating the immune response as manifested by the reduced levels of IgG2b and CD138 expressing cells. EPO treatment was associated with normalized IL-6 and RORγτ transcript levels as well as increased percentage of macrophages in the BM, and *in vitro* phagocytic activity of BMDM. On the other hand, EPO induced bone loss in MM mice to a similar extent as in healthy animals. This effect is associated with EPO- and MM-related changes in the expression of RANKL and OPG in the BM.

## DISCUSSION

After more than a decade in which rHuEPO became a widely used therapeutic agent for the anemia, several recent reports suggested a detrimental effect of the hormone on cancer evolution, which led to a significant drop in its clinical application^40^. This led to the current joint ASCO/ASH guidelines regarding the use of ESAs in clinical practice^41^. We and others have reported on longer survival and improved immune parameters in ESA-treated (for anemia) MM patients^6^,^7^,^12^,^13^,^14^,^15^,^19^. In that respect, ESA are still widely used to treat the anemia associated with MM^2^. However, reports suggesting shorter survival in ESA-treated MM patients were also published^20^. The present study focused on the 5T33MM mouse model, to examine, in parallel, the effects of EPO on the different cell systems involved in MM. The 5T33MM model is characterized by a compromised immune status before the bone loss becomes evident^36^,^37^. It thus enables to test the assumption that EPO improves the immune response to MM but may accelerate the development of the associated bone disease. This mouse model is therefore a proper choice as it prevents the superimposition of the bone loss induced by EPO and that already caused by the disease. While in patients EPO is typically administered for the anemia associated with the disease, in the 5T33MM mouse model we did not account for Hgb levels at the time of EPO administration. Yet, similar to the case in patients, the MM mice displayed decreased Hgb levels (on day 38 post MM injection), which were corrected by EPO treatment. Our findings portray EPO as a pivotal regulator of the tight balance between hematopoiesis, immune potency and bone metabolism in the 5T33MM mouse model displaying immuno-stimulatory effects on one hand but enhancing bone loss on the other.

We have previously shown that EPO treatment in both 5T2 and 5T33MM mice induced tumor regression and prolonged survival of the 5T33MM mice^11^. Here we show that EPO treatment in 5T33MM was accompanied by a decline in IgG2b, splenic CD138 expressing cells, and IL-6 transcripts. The MM associated IgG2b and kappa light chain serum levels which increased considerably following injection of the MM cells^36^ (Fig. 1), were significantly decreased in MM mice that were treated with EPO, while the non-pathological lambda light chain levels and IgA, remained unchanged (Fig. 1a–d and, Figure S2, respectively). These findings are in accordance with Zhou *et al.*, who have demonstrated an EPO-associated reduction in the pathological immunoglobulin in the MPC-11 MM mouse model^18^. Notably, we have excluded that these effects are mediated *via* direct EPO action on the 5T33MM cells, as they do not express EPO-Rs (data not shown). In addition to the BM, the 5T33MM model was also shown to present in the spleen and in the liver^36^. We have found, that spleen weights of the 5T33MM mice increased by 7-fold (p < 0.001) compared to healthy mice (data not shown). EPO treatment in the 5T33MM mice led to a reduction in CD138 expressing cells, mirroring the decrease in the levels of the pathological immunoglobulin. This is in contrast to the EPO-induced increase in the levels of plasma cells in EPO treated healthy mice, which may indicate EPO-improved immunological functions.

We show that 5T33MM mice displayed an elevation in IL-6 transcripts, which was decreased upon EPO treatment (Fig. 1g). The lack of effect of EPO treatment on IL-6 levels in BM of healthy mice supports the notion that the decrease in IL-6 is not caused by an EPO responsive cell (e.g. erythroblasts^4^, dendritic cells^9^, macrophages^10^), but rather an indirect effect on the 5T33MM cells that express less IL-6 in response to signals emanating from other EPO responsive cells. Our findings herein are in line with the EPO-related decrease in IL-6, observed in the MPC-11 mouse model^18^, and with previous findings on reduced IL-6 levels in EPO treated MM patients^7^,^42^. EPO-mediated reduction of IL-6 levels in several MM constellations (murine MM models and MM patients) validates this conclusion. Notably, others have found that EPO treatment had no effect on IL-6 serum levels in MM patients^43^. IL-6 plays a critical role in the balance between Th17 and Treg cells. IL-6, together with TGF-β, induces the development of Th17 cells from naïve T cells; in contrast, it inhibits TGF-β-induced Treg differentiation^44^. In the current study, we did not detect significant changes in transcript levels of TGF-β in the BM of EPO treated MM mice compared to diluent-injected MM mice, and healthy controls (data not shown).

Elevated Th17-associated cytokines were found to promote the proliferation of MM cells and to inhibit Th1-associated responses^45^. Here, we demonstrate for the first time, an elevation in RORγτ and FOXP3 transcription factors expressed in Th17 and Treg cells, respectively, in BM of 5T33MM mice, alongside an increase in the respective cytokines, IL-17A and IL-10 (Fig. 2). The issue of Treg and Th17 cell levels in MM patients is still controversial^23^. Our data on the increase in Treg-associated parameters are in line with studies demonstrating an increase in Treg cells in BM of 5T2 MM mice^46^. EPO treatment to the 5T33MM mice reduced RORγτ but had no effect on FOXP3 expression levels. Along these lines, an EPO-mediated reduction in Th17 cells was also presented in an experimental autoimmune encephalomyelitis mouse model^47^. In this case, disease was not associated with an increase in the Treg cell population, and Treg numbers were elevated by EPO^47^. The EPO-associated decrease in IL-6 transcripts in BM from MM mice, is in line with the inhibition of Th17 but not of Treg cells. The reported effects on the MM-associated increase in the proinflammatory cytokine IL-17A^48^ and the positive effect of this cytokine on MM growth^45^, lend support to the notion that EPO-mediated reduction in Th17 parameters contributes to the attenuation of MM. We thus propose that EPO treatment in MM attenuates disease progression by shifting the balance towards anti-inflammatory activity^47^.

We found that EPO treatment in the MM mice led to an elevation of IFN-γ transcripts in the BM. As MM is typically considered a Th2-mediated disease^49^, the elevation in IFN-γ transcripts, may point to attenuated MM progression by EPO mediated up-regulation of Th1 response. It appears that the presence of MM cells is necessary for the EPO mediated increase in IFN-γ, as EPO treatment in itself did not change the levels of IFN-γ. In that respect, we have found that isolated BMDM, treated with EPO do not respond by increase in IFN-γ (data not shown). Hence, the identity of the cells responding to EPO by increasing IFN-γ, in context of MM, remains to be elucidated. Noteworthy, potential cell candidates that express IFN-γ in response to direct or indirect effects of EPO include a variety of cells as T cells, B cells, NK cells, and antigen presenting cells^50^.

The lack of EPO effect on IL-6 and IFN-γ transcripts in diluent-treated healthy mice supports the notion that the EPO-mediated effects in MM, are conferred *via* EPO-R expressing cells as direct and/or indirect pathways involving MM.

The observed reduction in macrophage (F4/80^+^CD11b^+^) counts in the BM of MM mice (Fig. 3b) could reflect changes in the macrophage profile associated with MM 32. While EPO treatment in healthy mice increased the levels of splenic macrophages (F4/80^+^CD11b^+^)^10^, it did not affect the levels of F4/80^+^CD11b^+^ cells in the BM.

We have recently shown^30^ that EPO treatment in mice increased the level of monocyte-derived cells in the BM, potentially representing an EPO responsive population of macrophage and osteoclast precursors in the BM. In 5T33MM mice, EPO treatment was accompanied by increased levels of BM macrophages. Recent clinical studies suggest that macrophages play a critical role *in vivo*, in protecting myeloma cells from chemotherapy-induced apoptosis^51^,^52^. In this respect, it was reported that EPO mediated the production of pro-angiogenic factors from macrophages in MM patients^53^. Our current finding that EPO enhanced the capacity of BMDM to phagocytose 5T33MM cells (Fig. 4), suggests that alongside MM promoting actions of EPO on macrophages, it may also be beneficial in stimulating anti-MM actions of the macrophages. Interestingly, EPO action on the BMDM was accompanied by an increase in surface expression levels of IFN-γR (Fig. 4d,e), though it did not lead to an increase in IFN-γR transcripts (data not shown), suggesting other possible mechanisms of regulation.

Contrasting the beneficial effects of EPO on immune-associated parameters, we found that EPO treatment enhanced bone loss in the MM mice.

In a recent review, the International Myeloma Working Group updated the diagnostic criteria for MM^54^.The authors call to attention the distinction between the detection of osteolytic focal lesions and the manifestation of generalized osteoporosis. The updated guidelines recommended the exclusion of osteoporosis from the diagnostic criteria because generalized osteoporosis is often not pathognomonic to MM.

Moreover, MM can be diagnosed even in the absence of osteolytic focal lesions. For instance, the detection of clonal BM plasma cells above 10% together with hypercalcemia, renal insufficiency or anemia will lead to the diagnosis of MM. While the alternative 5T2MM model does present osteolytic focal lesions and a mild generalized bone loss, the 5T33MM mice employed in this study are also a suitable MM model. In terms of plasma cell malignancy, the latter displays a more aggressive picture and the absence of bone lesions may result from the 3–6 times faster progression of the disease than in the 5T2MM model^36^, leading to animal death before the development of detectable bone loss. Although no generalized bone loss was observed based on the microtomographic analysis of the distal femur, the elevated RANKL/OPG ratio in the BM microenvironment in the MM mice, support the notion of induced focal osteolysis (Fig. 6c). This is in line with previous observations (reviewed in^55^), and it is therefore likely to assume that with time, bone lesions could become apparent in the 5T33MM mice. Interestingly, the effect of EPO presented a more complex picture; EPO induced RANKL expression in healthy mice but did not further increase the already elevated levels in the MM mice (Fig. 6). This non-additive effect supports the opposite effect of EPO on MM progression and bone loss. In MM patients, elevation of RANKL is considered as a prognostic marker for MM survival^56^. Our findings prompt us to assume that on one hand, EPO attenuates MM progression, which would lower RANKL expression, but on the other hand, EPO stimulates bone resorption *via* increased RANKL expression. The unchanged levels of RANKL in response to EPO in MM mice are therefore the net result of these two antagonizing actions of EPO. Our finding that OPG was higher in the EPO-treated groups, despite the actual bone loss, suggests that EPO does not directly regulate OPG levels. We thus postulate that the observed increase in OPG is a compensatory mechanism aimed at attenuating the EPO-induced stimulation of bone resorption. Coupling between bone resorption and formation implies that as EPO directly stimulates osteoclasts^30^, the expression of anti-osteoclastogenic signals (e.g. OPG) increases in osteoblasts. Similar response has been reported in other conditions such as Wilson Disease osteopathy^57^, and post-menopausal osteoporosis^58^. Further studies may be warranted to test whether OPG and/or RANKL/OPG ratio can still be used as markers of bone resorption in MM.

The opposing effects of EPO on the immune and skeletal system in 5T33MM, demonstrated in the current study, thus reveal that EPO may act as a double-edged sword, by stimulating the immune response leading to attenuated MM progression, while accelerating bone resorption, possibly *via* its direct action on BM monocyte-derived cells. Clinically, this finding portrays a new angle of complexity to the decision making process regarding EPO treatment in MM, as well as in other clinical conditions where EPO is the treatment of choice for the accompanying anemia. A better understanding of the osteoimmunological roles of EPO may advocate for the use of rHuEPO along with targeted bone protective treatment in MM patients, to attenuate the anemia and MM progression, while also preventing bone damage. The bone protective treatment may target the elevated RANKL levels (e.g. RANKL blocking antibodies (denosumab), or other agents directly inhibiting osteoclasts (e.g. bisphosphonates)^59^. The advent of bone anabolic agents e.g. anti Dkk-1, anti sclerotin and teriparatide may also ameliorate adverse effects on bone disease rendering EPO treatment still worthwhile^60^. An alternative to EPO treatment is the action-A decoy receptor, ACE-011 (sotatarcept) which not only has bone anabolic properties but also has positive effects on hematocrit^61^.

The vast majority of MM patients currently receive either imunomodulatory agents (IMIDs) or proteasome inhibitors as first line therapy^62^. Higher incidence of venous thromboembolism (VTE) was reported in MM in general, but it was aggravated by the use of IMIDs^63^,^64^,^65^. Moreover, the combined use of IMIDS with EPO might increase the risk of VTE^66^. On the other hand, it should be emphasized that proteasome inhibitors have been shown to be safe, even when combined with ESAs, as shown in the VISTA trial^21^.

In practice, basic research projects such as the current one must be followed by clinical trials. Future research beyond this study will address whether the crosstalk of erythroid stimulating agents with any of the currently-used drugs for MM and MM-associated bone disease could be clinically beneficial. We expect that this knowledge will bear significant relevance to MM patients, as well as to a diverse population of patients treated with ESAs.

## Materials and Methods

### Animals and rHuEPO injections

Mouse handling and the experimental procedures were approved by the Institutional Animal Care and Use Committee of the Tel-Aviv University (permit number: M-12-031) and were performed in accordance with the approved guidelines.

The 5T33MM cell line was generously provided by Prof. Vanderkerken (Universiteit Brussel). This study was performed on 8–10 weeks old female C57BL/KaLwRij mice (Harlan, CPB Zeist, Netherlands). Mice were injected intravenously (i.v.) with 5 × 10^4^ 5T33MM cells and 8 days later, subcutaneously (s.c.) with 30U rHuEPO (Epoetin α, Eprex^®^, Janssen), or diluent (saline) for 10 consecutive days followed by 3 injections per week for 3 weeks as previously described^11^. Data analysis was performed from three experiments each containing n≥5 mice in each group. 5T33MM mice were sacrificed on day 38 following 5T33MM injection.

### ELISA

Sera were diluted 1:30,000 or 1:4 and analyzed for IgG2b or IgA levels, respectively, by ELISA kits (Bethyl Laboratories) according to the manufacturer’s instruction. Plates were read at 450 nm in an ELISA SpectraMAX 190 microplate reader.

### Western blot analysis

Serum samples (0.5 μL) were separated on 10% SDS-PAGE and transferred to a nitrocellulose membrane. The nitrocellulose membrane was incubated with Rabbit anti-Mouse kappa light chain (ICN Biomedicals, USA) or with Rabbit anti-Mouse lambda light chain antibodies (ICN Biomedicals, USA) diluted 1:500, followed by peroxidase labeled Goat anti-Rabbit antibodies (Dako, Denmark) diluted 1:150. Light chain levels were normalized to serum albumin as detected by Goat anti-Mouse serum albumin (Abcam, Atlanta, USA) diluted 1:1000, followed by IRDye^®^ 800CW conjugated Donkey anti-Goat IgG (H+L) (LI-COR Biosciences, Nebraska,USA) diluted 1:5000.

### CD138 fluorescence staining

Paraffin-embedded sections of spleens were de-paraffinized with Xylene and hydrated in declining concentrations of ethanol. Slides were then rinsed in PBS and covered with Proteinase K (20 mg/ml in 50 mM Tris, 1 mM EDTA, 0.5% Triton X100, pH = 8.0) for 3 min at RT. Sections were blocked with 2% Goat serum for 30 min at RT and stained overnight at 4 °C, with Rat anti Mouse CD138 antibody (Biolegend, San Diego, CA, USA) diluted 1:400, followed by Goat anti-Rat IgG (H+L), Alexa Fluor^®^ 555 (Thermo Fisher Waltham, MA, USA) diluted 1:500 and DAPI (1 μg/μl) for 1 hour at RT. Coverslips were mounted and the images were viewed with a  ×  63/1.4 oil objective, using a Leica TCS SP5 microscope and Leica SP5 software (LAS-AF, Leica, Germany).

### Flow Cytometry analyses

BM cells were flushed from femur and tibia. An aliquot of 1 × 10^6^ cells was stained with the following fluorescence-conjugated antibodies: Rat anti-Mouse F4/80 FITC, Rat IgG2aκ (isotype control), Rat anti-Mouse CD11b PE, Rat IgG2bκ (isotype control), all from eBiosciences, San Diego, CA, USA. Cells were incubated with the antibodies for 30 min at 4 °C and washed with phosphate buffered saline (PBS) containing 2% fetal bovine serum (FBS). Cells were fixed with 1% paraformaldehyde (PFA)+PBS and analyzed on a FACSort flow cytometer (Becton-Dickinson, New Jersey, USA). Results were analyzed using Kaluza software (Beckman Coulter, Nyon Switzerland).

### qPCR

Total RNA was extracted from whole BM, flushed from long bones and dissolved in Trizol Reagent (Invitrogen, Grand Island, NY, USA) according to the manufacturer’s instructions. cDNA was produced using High Capacity cDNA Reverse Transcription Kit (Invitrogen, Grand Island, NY, USA). qPCR was performed using TaqMan gene expression assay (Applied Biosystems, California, USA) or using Kapa SYBR Fast qPCR (Kapa Biosystems, California, USA). qPCR was performed on StepOnePlus real time PCR machine (Applied Biosystems, California, USA). Changes in relative gene expression were calculated using the 2^−ΔΔct^ method, normalizing to the house keeping gene, Hprt. Primer sequences (Hylabs, Israel) and probes (Applied Biosystems) used for amplification are depicted in Table 1.

### Generation of bone marrow derived macrophages (BMDM)

Preparation of BM cell cultures was performed as described^10^. Briefly, bone marrow cells were isolated from femurs and tibias. Cells were then incubated for 7 days and distributed to petri bacterial plates, the period required for bone marrow cells to differentiate to macrophages in Dulbecco’s Modified Eagle medium (DMEM), containing 10% heat-inactivated FBS and supplemented with 30% L-conditioned medium. The medium was replenished on the 4th day. The non-adherent cells were purged and the macrophages (adherent cells) were collected by treatment with 15 mM EDTA in PBS for 15 minutes at 4 °C, followed by PBS addition and gentle pipetting. The macrophage population (bone marrow derived macrophages; BMDM), as assessed by expression of the F4/80 surface molecule by flow cytometry analysis, was typically 99% pure.

### Phagocytosis assay

BMDM (5 × 10^5^ cells) were incubated in 24 wells and stimulated in the presence or absence of EPO 5 U/ml for 24 h. 5T33MM (1.5 × 10^5^ cells) stained with carboxyfluorescein succinimidyl ester (CFSE) were added to each well. The plates were spun-down and incubated for 24 hours at 37 °C. Cells were collected, stained with anti-mouse CD11b PE (eBioscience) and analyzed by FACSort flow cytometery (Becton-Dickinson); phagocytosing BMDM were positive for both CFSE and PE. Phagocytosis was also assessed by microscopy using a Leica TCS SP5 microscope and Leica SP5 software (LAS-AF, Leica, Germany).

### IFN-γ receptor expression

BMDM (5 × 10^5^ cells) were incubated in 24 wells and stimulated in the presence or absence of EPO 5 U/ml for 24 h. Cells were collected and labeled with Rat anti-Mouse CD11b FITC and Hamster anti Mouse IFN-γ receptor 1 (IFN-γR) PE (eBioscience) and examined by Gallios flow cytometer and analyzed using Kaluza software (Beckman Coulter, Nyon Switzerland).

### Microcomputed tomography (μCT)

Femora (one per mouse) were examined as reported previously^30^, using the μCT50 system (Scanco Medical AG, Switzerland). Briefly, scans were performed at a 10-μm resolution. The mineralized tissues were segmented by a global thresholding procedure. Trabecular bone parameters were measured in the secondary spongiosa of the distal femoral metaphysis and cortical parameters were determined in the mid-diaphyseal region according to the standardized nomenclature.

### Statistical analysis

Values are expressed as mean ± SEM unless otherwise indicated. Student’s *t*-test (for pairwise comparison), or 1-way ANOVA with Bonferroni post-hoc test was used for multiple comparisons, using GraphPad Prism 7, San Diego CA, USA. Significant difference between groups was defined as p < 0.05.

## Additional Information

**How to cite this article**: Deshet-Unger, N. *et al.* Erythropoietin treatment in murine multiple myeloma: immune gain and bone loss. *Sci. Rep.* **6**, 30998; doi: 10.1038/srep30998 (2016).

## Supplementary Material

Supplementary Information

## Figures and Tables

**Table 1:** Primer sequences and probes used for PCR amplification.

Gene	Forward primer 5′ to 3′	Reverse primer 5′ to 3′
IFN-γ	TCAAAAGAGTTCCTTATGTGCCTA	TACGAGGACGGAGAGCTGTT
IL-17A	CAGGGAGAGCTTCATCTGTGT	GCTGAGCTTTGAGGGATGAT
IL-10	CAGAGCCACATGCTCCTAGA	GTCCAGCTGGTCCTTTGTTT
Hprt	TCCTCCTCAGACCGCTTTT	CCTGGTTCATCATCGCTAATC
RORγτ	Mm01261022	
FOXP3	Mm00475162	
IL-6	Mm00446190	
RANKL	Mm00441908	
OPG	Mm00435452	
Hprt	Mm00446968	
