# Supplementary material for: Erythropoietin treatment in murine multiple myeloma: immune gain and bone loss
Source: Sci Rep. 2016 Aug 2;6:30998. doi: 10.1038/srep30998 (PMC4969594; doi:10.1038/srep30998)
Supplement: Supplementary Information [file srep30998-s1.pdf]

Erythropoietin treatment in murine multiple myeloma: immune gain and bone loss

Naamit Deshet-Unger[1], Sahar Hiram-Bab[1,2], Yasmin Haim Ohana[1], Moshe Mittelman*[3], Yankel Gabet*[2] and Drorit Neumann*[1]

[1]Department of Cell and Developmental Biology, and [2]Department of Anatomy and Anthropology, Sackler Faculty of Medicine, Tel-Aviv University, Israel, [3]Department of Medicine, Tel Aviv Sourasky Medical Center, Sackler Faculty of Medicine, Tel-Aviv University, Israel
*equal contribution

Address correspondence to:
Drorit Neumann, Department of Cell & Developmental Biology
Tel: +972-3-6407256; Fax: +972-3-6407432; Email: histo6@post.tau.ac.il
Sackler Faculty of Medicine, Tel Aviv University
P.O. Box 39040, Tel Aviv 69978
Israel

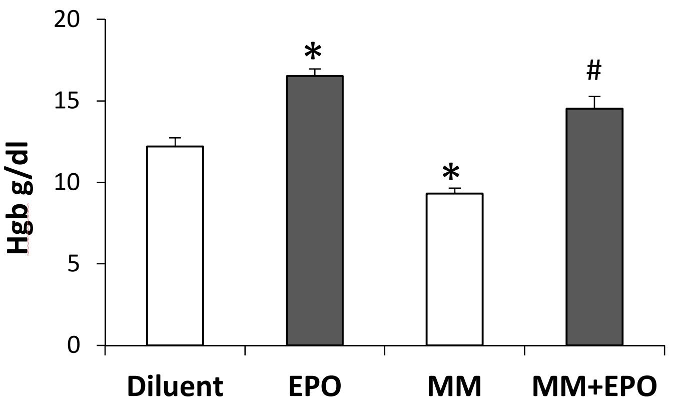

Figure S1. EPO treatment leads to increased hemoglobin (Hgb) levels. Sera from healthy or MM mice treated with EPO or diluent were subjected to blood test and Hgb levels were measured. Data are Mean±SEM, N>4; *MM *versus* Diluent; #MM+EPO *versus* MM, $p<0.05$, when analyzed by 1-way ANOVA with Bonferroni post-hoc test.

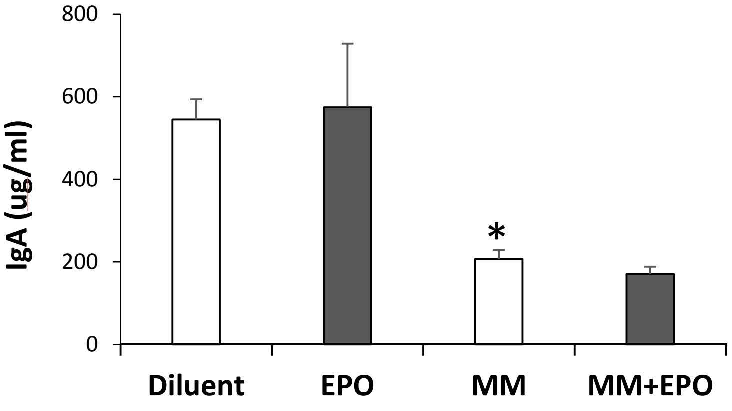

Figure S2. EPO treatment does not affect serum IgA levels. Sera from healthy or MM mice treated with EPO or diluent were subjected to ELISA analysis for IgA on day 28. Data are Mean±SEM; N>5 *MM *versus* Diluent, $p<0.05$, when analyzed by 1-way ANOVA with Bonferroni post-hoc test.
